# Model-based economic evaluation of pharmacopuncture for chronic low back pain: a 3-year Markov analysis

**DOI:** 10.3389/fpubh.2025.1710425

**Published:** 2026-01-13

**Authors:** Ye-Seul Lee, Sujin Kim, In-Hyuk Ha, Yoon Jae Lee

**Affiliations:** Jaseng Spine and Joint Research Institute, Jaseng Medical Foundation, Seoul, Republic of Korea

**Keywords:** acupoint injection therapy, chronic low back pain, cost-effectiveness, Markov modeling, pharmacopuncture

## Abstract

**Background:**

Chronic low back pain (cLBP) is the leading cause of disability worldwide and a major driver of healthcare costs. Excessive reliance on pharmacological treatments raises sustainability and safety concerns, highlighting the need for effective non-pharmacological alternatives. Pharmacopuncture (PPT), also known as herbal acupuncture or acupoint injection therapy, is widely practiced in some Asian countries (e.g., Korea and China) but remains uninsured in most health systems and largely excluded from international guidelines despite its clinical adoption. Evidence on its long-term cost-effectiveness compared with standard physiotherapy (PT) is limited.

**Objective:**

To evaluate the cost-utility of PPT versus PT for patients with cLBP using a Markov model designed in accordance with international pharmacoeconomic guidelines and standard government requirements for cost-effectiveness analysis.

**Methods:**

A three-state Markov model (mild, moderate, severe pain) projected outcomes over 3 years with 3-month cycles. Clinical inputs were derived from a multicenter pragmatic RCT. Costs were estimated from healthcare system, restricted societal, and full societal perspectives, incorporating medical, non-medical, and productivity loss costs estimated using national health databases. Quality-adjusted life years (QALYs) were calculated using EQ-5D-5L data. Incremental cost-effectiveness ratios (ICERs) were benchmarked against the Korean willingness-to-pay (WTP) threshold. Deterministic and probabilistic sensitivity analyses assessed robustness.

**Results:**

Over 3 years, PPT was less costly and more effective than PT. From the healthcare perspective, PPT reduced costs ($1,304 vs. $1,385) while yielding higher QALYs (2.30 vs. 2.23), resulting in dominance (ICER = −$1,145 per QALY). From the societal perspective, including productivity, PPT further strengthened its dominance ($25,760 vs. $31,962). Probabilistic sensitivity analysis showed a 97.7% (healthcare) and 99.4% (societal) probability of cost-effectiveness at the WTP threshold. Results were robust across sensitivity scenarios.

**Conclusion:**

This is the first model-based economic evaluation of pharmacopuncture for cLBP. Findings indicate that PPT can deliver higher QALYs at lower costs compared with PT, underscoring its potential as a high-value non-pharmacological alternative. This aligns with WHO priorities for evidence-based integration of traditional medicine, supports policy discussions on equitable coverage, and informs sustainable chronic pain management strategies across health systems.

## Introduction

Chronic low back pain (cLBP) is a leading cause of disability worldwide and a major driver of healthcare costs and productivity loss. In 2020, an estimated 619 million people were affected globally, with cLBP ranked as the top contributor to years lived with disability (1). The condition also generates substantial indirect costs through productivity loss, underscoring the need for effective and cost-efficient interventions. Contemporary guidelines increasingly recommend non-pharmacological strategies such as exercise therapy, multidisciplinary rehabilitation, and acupuncture as first-line options (2). Yet, uncertainty in the evidence base contributes to ongoing policy debates about the role of complementary and integrative modalities in reimbursement frameworks.

Pharmacopuncture (also termed acupoint injection therapy or herbal acupuncture) is an emerging TCIM intervention that combines mechanical stimulation of acupuncture points with localized delivery of herbal or bioactive solutions (2, 3). It is hypothesized to enhance and prolong the effects of conventional acupuncture (4). Widely practiced in Korea and China, acupoint injection therapies are increasingly integrated into Traditional Chinese Medicine (TCM) services (5). Evidence from pragmatic randomized controlled trials (RCTs) indicates benefits in musculoskeletal and spine disorders, including chronic low back pain (cLBP), with pharmacopuncture demonstrating superior short-term improvements in pain, function, and quality of life compared with physiotherapy (PT) (2, 6). Reflecting this, Korean Medicine clinical practice guidelines (CPGs) recommend pharmacopuncture for musculoskeletal conditions, and Chinese CPGs list acupoint injection as a TCM option with cautious endorsement.

In contrast, pharmacopuncture is absent from Western guidelines. The American College of Physicians (2017) recommends acupuncture (7) but not acupoint injections, and while Medicare covers acupuncture for chronic pain (8), injection therapies are excluded. The UK’s NICE guideline for low back pain and sciatica (NG59, 2016; updated 2020) does not recommend acupuncture for these conditions and accordingly omits related modalities such as pharmacopuncture, while NICE guideline for chronic pain recommends acupuncture for patients with chronic primary pain (9). The World Health Organization has encouraged research into and integration of traditional medicine, but has yet to issue specific guidance on pharmacopuncture. This omission is consistent with the limited long-term, high-quality evidence for acupoint injectable therapies. This divergence illustrates a persisting practice-policy gap: pharmacopuncture is widely used and guideline-endorsed in Asia yet remains excluded from reimbursement in Korea and absent from international guidelines (4, 10). Barriers include lack of standardized protocols, regulatory concerns about injectable herbal preparations, and limited long-term, large-scale evidence to substantiate cost-effectiveness and safety.

Economic evaluation is therefore critical to determining whether widely used but uninsured services provide value for money. Prior cost-utility studies suggest acupuncture and related interventions often achieve acceptable incremental cost-effectiveness ratios (ICERs). However, evidence specific to pharmacopuncture remains sparse and limited in duration. To address this gap, we conducted a cost-utility analysis comparing pharmacopuncture (PPT) with physiotherapy (PT) for cLBP using data from a multicenter pragmatic RCT in South Korea. A three-state Markov model projected costs and quality-adjusted life years (QALYs) over a 3-year horizon from both healthcare system and societal perspectives, consistent with Korean pharmacoeconomic guidelines. By estimating the ICER of PPT versus PT against the national willingness-to-pay threshold, this study seeks to inform clinicians, payers, and policymakers in Korea and globally considering whether pharmacopuncture merits integration into evidence-based guidelines and reimbursement frameworks.

## Methods

### Markov model

A three-state Markov model was utilized to evaluate the cost-utility of PPT versus PT for cLBP over a 3-year time horizon, with 3-month cycles. The model consisted of three mutually exclusive health states—mild, moderate, and severe pain—and simulated disease progression over a 3-year time horizon using 3-month cycles, consistent with international pharmacoeconomic modeling practice. Outcomes were expressed as incremental cost-effectiveness ratios (ICERs), defined as the additional cost per QALY gained by PPT relative to PT. ICERs were benchmarked against a willingness-to-pay (WTP) threshold of 30.5 million Korean Won (KRW, ≈26,647 USD) per QALY, in accordance with Korean pharmacoeconomic evaluation guidelines (11). Costs and health outcomes were discounted at 4.5% annually, with currency converted at the 2021 exchange rate of 1 USD = 1,144.61 KRW. Clinical inputs were derived from a multicenter pragmatic RCT conducted in South Korea (2).

### Model structure and clinical data

**Figure 1:**
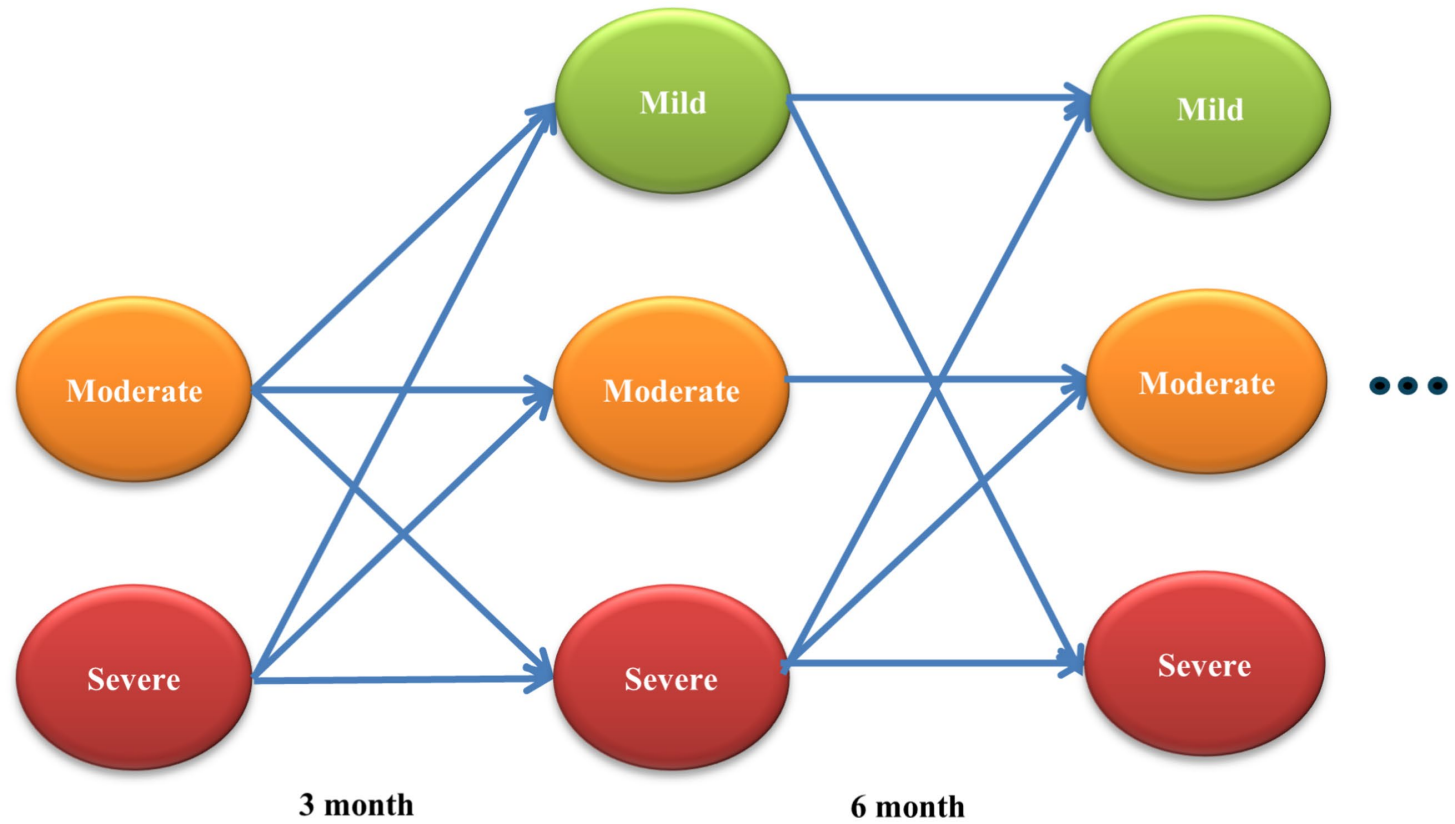
Schematic of the three-state Markov model used to evaluate pharmacopuncture (PPT) versus physiotherapy (PT) for chronic low back pain. Patients transition among mild, moderate, and severe pain states during each 3-month cycle over a 3-year time horizon. Arrows indicate all permitted transitions based on probabilities derived from the pragmatic randomized controlled trial. Background mortality was not included due to the young, working-age study population and absence of deaths in the trial.

The model structure is illustrated in Figure 1, which provides a schematic of the three health states and all possible transitions between them. Pain severity was categorized as mild (NRS < 4), moderate (4 ≤ NRS < 7), and severe (NRS ≥ 7). As the underlying pragmatic RCT (2) enrolled only patients with moderate or severe pain at baseline, initial states were restricted accordingly. The trial included 100 patients aged 19–70 with ≥6 months of cLBP and baseline NRS ≥ 5, randomized to 10 sessions of PPT or PT over 5 weeks (2, 12). Transition probabilities were estimated directly from observed changes in NRS categories at baseline, end of treatment, and 3-month follow-up, and these empirical estimates formed the basis for the transition matrices used in the Markov model.

### Model assumptions

Key assumptions are summarized in Table 1. The model applied fixed 3-month cycles over the 3-year horizon, with patients able to remain in or transition among mutually exclusive health states as permitted by the RCT-derived transition matrices. Patients in mild pain were assumed not to incur treatment costs, while those in moderate or severe pain received repeated treatment each cycle. Transition probabilities beyond 3 months were assumed constant, based on estimates from the final available follow-up (13). The probability of remaining in mild pain was assumed independent of treatment group, consistent with RCT findings. Background mortality was not modeled, as the study population consisted of working-age adults with no deaths reported in the study period and extremely low short-term mortality risk (2).

**Table 1:** Parameters used in the analysis.

Variable		Base-case	One-way sensitivity range^a^	Distribution parmeters^d^	Source
Assumption					
Discount rate (%)		4.5	3.5, 6.0	–	HIRA-guideline
Analysis period (year)		3	1, 5	–	–
Initial severity distribution		12.0: 88.0	± 0.03	–	Park et al. (2)
(Moderate: Severe, %)					
Medical cost for PPT group ($)					
Consultation		80.01	–	Fixed	HIRA-price index
Syndrome differentiation technique		17.42	–	Fixed	
PPT therapy		201.34	–	Fixed	
Type of PPT*		1	–	Fixed	HIRA-NPS
Medical cost for PT group ($)					
Consultation		107.53	–	Fixed	HIRA-price index
Scenario 1: (Superficial Heat Therapy + TENS) or (Superficial Heat Therapy + ICT)		43.83	–	Fixed	
Scenario 2: (Ultrasound + TENS) or (Ultrasound + ICT)		47.23	–	Fixed	
Scenario 3: (Superficial Heat Therapy + Laser Therapy)		61.40	–	Fixed	
Scenario 4: (Ultrasound +Laser Therapy)		64.80	–	Fixed	
Ratio of physiotherapy combinations (%)	Scenario 1	25	100, respectively	Beta (62,183, 186,549)	HIRA-NPS
	Scenario 2	61	100, respectively	Beta (151,727, 97,005)	
	Scenario 3	8	100, respectively	Beta (19,899, 228,833)	
	Scenario 4	6	100, respectively	Beta (14,924, 233,808)	
Non-medical cost for PPT group($)					
Transportation^c^		4.32	± 0.43	Gamma (0.43)	NECA-guideline
Time		183.83	± 12.15	Gamma (12.15)	Park et al. (2); HIRA, 2021; MOEL, 2021; Statistics Korea, 2021
Non-medical cost for PT group($)					
Transportation^c^		5.46	± 0.55	Gamma (0.55)	NECA-guideline
Time		209.40	± 14.14	Gamma (14.14)	Park et al. (2); HIRA,2021; MOEL,2021; Statistics Korea,2021
Productivity loss					
Productivity loss (per cycle, $)	Mild	1,645.46	± 123.74	Gamma (123.74)	Park et al. (2); HIRA, 2021; MOEL, 2021
	Moderate	2,585.98	± 92.96	Gamma (92.96)	
	Severe	3,405.16	± 108.29	Gamma (108.29)	
Utility (EQ-5D-5L)					
Mild (QALY^b^)		0.837 (0.209)	± 0.003	Beta (3009.990, 11377.064)	Park et al. (2)
Moderate (QALY^b^)		0.782 (0.195)	± 0.003	Beta (3738.664, 15392.143)	
Severe (QALY^b^)		0.750 (0.187)	± 0.003	Beta (2683.764, 11641.459)	

a These items are applied 95% CI of SE for one-way sensitivity range.

b The quality-of-life assessment score represents the final utility weight for each health condition. QALY was calculated over one cycle (3 months), and a sensitivity analysis was conducted on the QALY values.

c Due to difficulties in assuming a distribution, values from the literature were utilized with a 10% assumption.

d Beta distribution (alpha, beta) and gamma distribution (SE) values are presented.

CI, confidence interval; HIRA, Health Insurance Review and Assessment Service; ICT, interferential current therapy; MOEL, Ministry of Employment and Labor; NECA, National Evidence-based Healthcare Collaborating Agency; PPT, pharmacopuncture therapy; PSA, probabilistic sensitivity analysis; PT, physical therapy; QALY, quality-adjusted life year; TENS, transcutaneous electrical nerve stimulation.

### Target population, intervention, and comparator

The modeled population reflected the RCT sample: Korean adults aged 19–70 with cLBP of at least 6 months’ duration and baseline NRS ≥ 5 (2). This population is representative of patients typically treated in Korean medicine outpatient settings. PPT was defined as ten sessions administered twice weekly over 5 weeks. Protocols including pharmacopuncture solution, acupuncture points, and injection dosage, were assumed to be determined individually by licensed Doctors of Korean Medicine based on clinical assessment.

PT comparators were identified using the Korean Health Insurance Review and Assessment Service–National Patient Sample (HIRA-NPS). Modalities included deep heat therapy, superficial heat therapy, transcutaneous electrical nerve stimulation (TENS), and intermittent pelvic traction. Specific modalities, sites and duration were assumed to be physician-determined, with treatment frequency matched to PPT for comparability.

#### Transition probabilities

Transition probabilities were estimated from RCT data, which included baseline, post-treatment, and 3-month follow-up NRS scores (Table 2). Because the trial enrolled only moderate and severe cases, probabilities for mild pain were determined separately during model construction. No significant differences were observed between PPT and PT in the probability of remaining in mild pain, so pooled estimates were applied. For moderate and severe states, assigned treatments were re-administered each cycle.

**Table 2:** Transition probabilities.

From	To	Probability	One-way sensitivity range^a^	Distribution parmeters^b^	Source
Transition Probabilities for PPT group					
Mild (2-cycle)	Mild	0.731	± 0.081	Dirchlet (20.995, 7.686, 0.022)	Park et al. (2)
	Moderate	0.268	± 0.081		
	Severe	0.001	± 0.01		
Moderate	Mild (1-cycle)	0.472	± 0.195	Dirchlet (2.614, 1.618, 1.034)	
	Moderate	0.334	± 0.195		
	Severe	0.194	± 0.157		
Severe	Mild (1-cycle)	0.612	± 0.079	Dirchlet (22.413, 11.299, 3.048)	
	Moderate	0.321	± 0.078		
	Severe	0.067	± 0.037		
Transition probabilities for PT group					
Mild (2-cycle)	Mild	0.731	± 0.081	Dirchlet (20.995, 7.686, 0.022)	Park et al. (2)
	Moderate	0.268	± 0.081		
	Severe	0.001	± 0.01		
Moderate	Mild (1-cycle)	0.052	± 0.121	Dirchlet (0.125, 3.198, 0.954)	
	Moderate	0.765	± 0.186		
	Severe	0.183	± 0.155		
Severe	Mild (1-cycle)	0.209	± 0.071	Dirchlet (6.572, 20.097, 13.346)	
	Moderate	0.569	± 0.082		
	Severe	0.222	± 0.053		

a These items are applied 95% CI of SE for one-way sensitivity range.

b Dirichlet distribution (alpha) values.

### Cost

Costs were estimated in 2021 KRW and converted to USD using the 2021 average exchange rate (1 USD = 1,144.61 KRW). In accordance with Korean pharmacoeconomic evaluation guidelines, costs were estimated from three perspectives, each defined by distinct components: healthcare system, restricted societal, and full societal. The healthcare system perspective included only direct medical costs, such as consultation fees, syndrome differentiation techniques (for PPT), pharmacopuncture administration costs, and physiotherapy modalities. The restricted societal perspective included transportation and time costs in addition to all direct medical costs, while full societal perspective additionally incorporated productivity losses, valued using the human capital approach, based on Work Productivity and Activity Impairment (WPAI) data collected during the RCT and national wage statistics. This distinction reflects Korean pharmacoeconomic guidelines; the restricted perspective captures patient opportunity costs of treatment, while the full perspective estimates the broader macroeconomic burden of productivity loss.

PPT costs were micro-costed per session; PT costs were based on HIRA-NPS physiotherapy combinations. Transportation and time costs were derived from national surveys. Productivity losses, measured using WPAI during the RCT, were extrapolated across the 3-year horizon due to absence of longitudinal data. Costs and outcomes were discounted at 4.5%. Only patients in moderate or severe states incurred treatment costs. Detailed inputs are shown in Table 1 and Supplementary Table S1.

### Utility

Utility values were derived directly from the validated Korean EuroQol 5-Dimension 5-Level (EQ-5D-5L) value set, applied to a Korean-translated version of the EQ-5D-5L responses collected at baseline, 6, 13, and 25 weeks in the RCT. Utilities were mapped to the three pain severity states (mild, moderate, severe) defined using NRS, and QALYs were calculated using the area-under-the-curve method, applying the trapezoidal rule over 3-month cycles. As no significant between-group differences were observed for utilities within pain categories, pooled state-specific utilities were used for both groups (Table 1).

### Base-case analysis

The base-case analysis (Table 3) estimated the total costs and QALYs for PPT and PT over 3 years. Incremental costs and QALYs were derived by comparing PPT to PT, and the incremental cost-effectiveness ratio (ICER) was reported as cost per QALY gained. Details are provided in Supplementary Table S2.

**Table 3:** Base-case analysis.

Comparison	Total cost ($)	Incremental cost ($)^a^	Total effectiveness (QALY)	Incremental effect (QALY)^a^	ICER ($/QALY)
Healthcare system perspective					
PPT	1304.33	−80.19	2.3	0.07	Dominant (−1145.57)
PT	1384.52		2.23		
Societal system perspective					
PPT	25760.12	−6201.83	2.3	0.07	Dominant (−88587.57)
PT	31961.95		2.23		

a The incremental value was determined by subtracting the total value of PT from the total value of PPT.

ICER, incremental cost-effectiveness ratio; QALY, quality-adjusted life year; PPT, pharmacopuncture therapy; PT, physiotherapy.

### Uncertainty analysis

To assess the robustness of the model results, deterministic (one-way) and probabilistic sensitivity analyses (PSA) were conducted.

#### One-way deterministic sensitivity analysis

One-way deterministic sensitivity analysis was performed by varying key input parameters across plausible ranges, including the 95% confidence intervals or ±10% deviations from base-case values, where applicable. Parameters included discount rates (ranging from 3.5 to 6%), initial pain severity distribution, time horizon, medical costs, non-medical costs (e.g., transportation and time), utility weights, and transition probabilities (Supplementary Table S3). The impact of each parameter on the incremental cost-effectiveness ratio (ICER) was evaluated, and results were presented using tornado diagrams to identify the most influential variables. Net monetary benefit (NMB) was also calculated at the WTP threshold.

#### Probabilistic sensitivity analysis (PSA)

Probabilistic sensitivity analysis (PSA) was conducted to account for joint uncertainty across all model parameters. A Monte Carlo simulation with 1,000 iterations was implemented, drawing random samples from beta distributions for probabilities and utilities (bounded between 0 and 1), gamma distributions for cost parameters (reflecting right-skewed cost data), and Dirichlet distributions for transition probabilities across health states. Transportation costs were modeled with a fixed standard error of 10%. Results were presented on a cost-effectiveness plane as cost-effectiveness acceptability curves (CEACs).

## Results

### Base-case analysis

Over the 3-year horizon, PPT was both more effective and less costly than PT. From the healthcare perspective, mean costs per patient were $1,304.33 for PPT and $1,384.52 for PT, with an incremental cost of $80.19. QALYs gained were 2.30 and 2.23, respectively, yielding an incremental benefit of 0.07 QALYs. The corresponding ICER was negative ($-1,145.57 per QALY), indicating that PPT was the dominant strategy over PT. From a societal perspective, which incorporates transportation, time, and productivity loss, costs were $25,760.12 for PPT and $31,961.95 for PT, indicating that PPT remained a dominant strategy, with consistent cost-effectiveness under both perspectives.

### One-way sensitivity analysis

**Figure 2:**
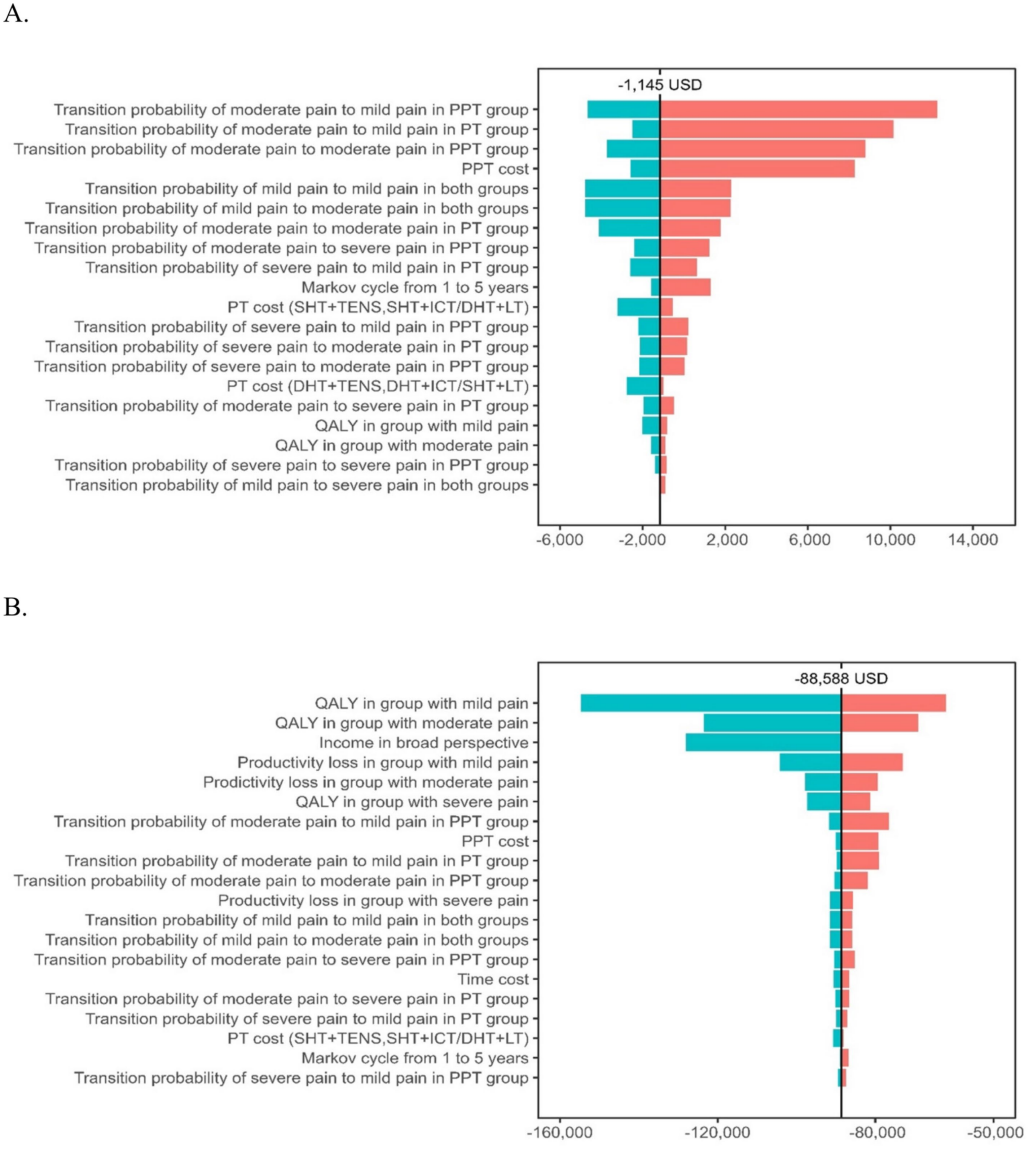
One-way sensitivity analysis tornado diagram. **(A)** Healthcare system perspective. **(B)** Societal perspective.

Figure 2 presents the 20 most influential parameters, ranked in descending order of impact on the ICER for PPT versus PT. Across all univariate scenarios, the ICER for PPT remained well below the WTP threshold of 30.5 million KRW (≈26,647 USD). The range of ICERs ranged from 6,652 to 22,127 USD per QALY across the full range of inputs tested, consistently supporting cost-effectiveness. The most influential parameters were the utility weight for moderate pain, the cost of PPT, and the transition probability from severe to moderate pain. Variations in non-medical costs and productivity loss also affected results but did not alter the cost-effectiveness conclusion, confirming that PPT remains a cost-effective strategy across a wide range of plausible assumptions. Detailed numerical results are provided in Supplementary Table S3.

### Probabilistic sensitivity analysis

**Figure 3:**
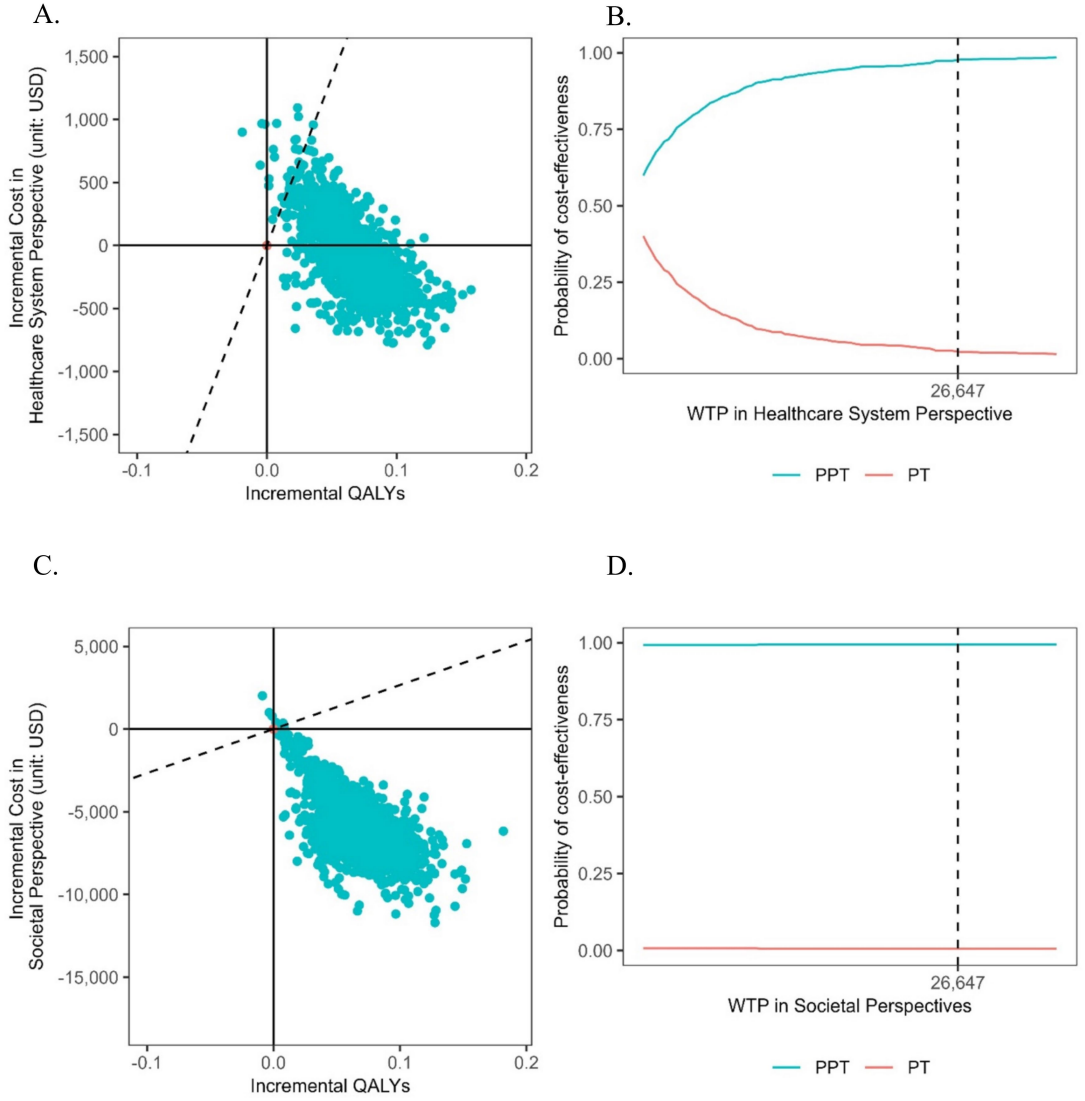
Probabilistic sensitivity analysis. **(A)** Scatterplots for the incremental cost-effectiveness ratio (ICER) in healthcare perspective. **(B)** Cost-effectiveness acceptability curve in healthcare perspective. **(C)** Scatterplots for the incremental cost-effectiveness ratio (ICER) in societal perspective. **(D)** Cost-effectiveness acceptability curve in societal perspective.

PSA results summarized in Figure 3 reinforced the robustness of the findings. From the healthcare system perspective, the average cost of PPT was $1,299.99 (SD = $300.71), while that of PT was $1,360.74 (SD = $202.83). At our pre-defined WTP threshold, the probability that PPT is cost-effective reached 97.7%, indicating strong confidence in its economic value. From the restricted societal perspective, the average cost of PPT was $25,686.20 (SD = $1,794.86), compared to $31,533.64 (SD = $1,775.58) for PT. At the same WTP threshold, the cost-effectiveness probability of PPT rose to 99.4%, underscoring its dominant performance when broader economic considerations are included.

## Discussion

This study is the first economic evaluation to assess the cost-utility of PPT versus PT for cLBP in South Korea using a Markov model informed by a multicenter pragmatic RCT. Over a 3-year horizon, PPT was consistently more effective and less costly, dominating PT from both healthcare and societal perspectives. Sensitivity analyses including probabilistic simulations confirmed the robustness of these findings, with PPT showing a 97.7–99.4% probability of cost-effectiveness at the Korean WTP threshold. These results provide strong evidence that PPT may represent a high-value treatment alternative for cLBP in clinical practice settings where complementary and integrative medicine modalities are provided.

Compared to our previous within-trial analysis (12) which was limited to a 25-week horizon, this study extended the timeframe to 3 years and incorporated transition dynamics between three clinical relevant states (mild, moderate, severe pain). The current findings reaffirm the earlier conclusion that PPT is both more effective and less costly than PT, but further demonstrates its robustness under repeated treatment cycles and broader perspectives. By modeling direct, indirect, and productivity costs, this study better reflects real-world treatment patterns and long-term burden.

In interpreting these findings, it is important to note that PPT is currently provided as an out-of-pocket service in Korea, whereas PT is reimbursed under the NHIS (6). This contrast reflects the most recent clinical guidelines, which prioritize non-pharmacological therapies as first-line management (7), and underscores a key policy question: whether an uninsured but widely used TCIM intervention offers sufficient value to merit broader integration alongside reimbursed conventional options. Despite its wide use in some Asian countries and its endorsement in Korean and Chinese clinical practice guidelines, PPT remains largely absent from global guidelines of which the recommendations are generally limited to acupuncture. For example, Medicare in the United States reimburses acupuncture for chronic pain, while in the United Kingdom, NICE supports acupuncture for chronic primary pain but not for low back pain or sciatica. These omissions underscore the need for more rigorous, long-term evaluations of nonpharmacological therapies, including pharmacopuncture, to establish their sustained effectiveness and cost-effectiveness. By providing economic evidence from a 3-year model, our study contributes to closing this gap, but further confirmatory research is essential to guide reimbursement and guideline development both in Korea and internationally.

Out results echo a broader body of health economic evidence supporting integrative therapies. For example, a recent 10-year Markov analysis compared integrated care of acupuncture and usual care to usual care only for knee osteoarthritis and found an ICER of about $11,100 USD per QALY, which was well below a $20,000 WTP threshold (14). Similarly, a 5-year model in chronic low back pain showed that acupuncture added to standard care improved QALYs at modest incremental cost, with an ICER around $2,900 USD per QALY (15). These findings align with broader evidence that many non-pharmacologic integrative therapies for chronic pain yield ICERs below common cut-offs which is often under $50,000/QALY, and some even produce net savings when societal costs are considered (16).

Integrative therapies for neurologic and other chronic conditions have consistently demonstrated favorable cost-effectiveness in diverse settings. Recent economic evaluations of PPT underscore this trend. In South Korea, a pragmatic trial found pharmacopuncture for chronic neck pain yielded slightly higher QALYs and lower overall costs compared to usual care (17). Similar findings are emerging elsewhere. For example, in China adding an herbal injection therapy to standard care for chronic stable angina produced better outcomes at an acceptable incremental cost (ICER ≈ $29,589 per QALY), falling below cost-effectiveness thresholds (18). This aligns with earlier analyses showing acupuncture or herbal add-ons can yield net economic gains, as seen in migraine prevention in Taiwan where acupuncture outperformed drug prophylaxis (19), or in COPD management in China where herbal granules plus usual care achieved an ICER around $300 per QALY (20). In summary, long-term modeling and trial data suggest that integrative medicine approaches often offer strong economic value across conditions from arthritis and back pain to migraines, cardiovascular and respiratory diseases, and even cancer pain (14, 19). This convergence of evidence strengthens the case that incorporating treatments like acupuncture, herbal medicine, or pharmacopuncture can be a cost-effective component of chronic disease management. Importantly, such modalities may help mitigate excessive medication use by providing effective non-pharmacologic options, addressing a key concern in chronic care long-term drug side effects including opioids overuse and polypharmacy (21).

Several limitations warrant consideration. Transition probabilities were derived from a single pragmatic RCT of 100 patients, and assuming constancy beyond the observed period may oversimplify the fluctuating natural course of cLBP. Extrapolation of short-term WPAI productivity data over 3 years may not fully capture workforce dynamics. The trial population was limited to Korean adults aged 19–70, which constrains generalizability to older patients or health systems with different cost structures. Moreover, PPT protocols in the trial were individualized, whereas policy adoption would likely require standardized formulations and procedures to ensure safety, reproducibility, and regulatory compliance. Future studies with larger, more diverse cohorts, longer follow-up, and external validation will be essential to strengthen the evidence base.

In conclusion, this study extends prior evidence that pharmacopuncture is not only clinically effective for chronic low back pain but also cost-effective over a longer horizon, outperforming physiotherapy across all perspectives examined. The extended 3-year Markov model demonstrates the economic and clinical value of PPT in a real-world setting, reinforcing earlier within-trial results and aligning with international evidence supporting integrative therapies for chronic conditions. These findings support broader inclusion of PPT in clinical guidelines and reimbursement policies for cLBP and suggest that well-designed integrative interventions can offer high-value care. Further research is warranted to validate these results in diverse populations, longer timeframes, and through health system implementation studies.

## Data Availability

The original contributions presented in the study are included in the article/Supplementary material, further inquiries can be directed to the corresponding author.
