# Supplementary material for: Model-based economic evaluation of pharmacopuncture for chronic low back pain: a 3-year Markov analysis
Source: Front Public Health. 2026 Jan 13;13:1710425. doi: 10.3389/fpubh.2025.1710425 (PMC12850513; doi:10.3389/fpubh.2025.1710425)
Supplement: Supplementary file 1 [file Data_Sheet_1.docx]

**Supplementary Table 1. Cost components and data sources for cost estimation.**

| **Category** | **Cost component** | **Data source** |
| --- | --- | --- |
| **Medical cost** | **Treatment** | Health Insurance Review and Assessment Service (HIRA, 2021). Medical, Dental, and Korean Medicine Fee File.  Accessed August 06, 2024 (https://www.hira.or.kr/eng/main.do#) |
|  |  | Health Insurance Review and Assessment Service (HIRA, 2019). HIRA-NPS: National Patient Sample Data. |
|  | **Consultation** | Health Insurance Review and Assessment Service (HIRA, 2021). Price index: Medical, Dental, and Korean Medicine Fee File. Retrieved August 06, 2024 (https://www.hira.or.kr/eng/main.do#) |
| **Non-medical cost** | **Transportation** | National Evidence-based Healthcare Collaborating Agency (NECA, 2021). Methodological Guide for Economic Evaluation of Medical Intervention. (p.108) |
|  | **Time** | Park, K. S., et al. (2023). A pragmatic randomized controlled trial on the effectiveness and safety of pharmacopuncture for chronic lower back pain. Journal of Pain Research, 16, 2697–2712. |
|  |  | Statistics Korea. (KOSIS, 2021). Economically Active Population Survey. Accessed November 19, 2024 (https://kosis.kr) |
|  |  | Healthcare Big Data Open System of Year 2021. 3-level Disease Statistics; Number of Patients by Gender/10-year Age Group. Accessed November 21, 2024 (https://opendata.hira.or.kr) |
|  |  | Ministry of Employment and Labor (MOEL, 2021). Survey Report on Labor Conditions by Employment Type. Accessed November 19, 2024 (https://kosis.kr) |
|  | **Productivity loss** | Park, K. S., et al. (2023). A pragmatic randomized controlled trial on the effectiveness and safety of pharmacopuncture for chronic lower back pain. Journal of Pain Research, 16, 2697–2712. |
|  |  | Healthcare Big Data Open System of Year 2021. 3-level Disease Statistics; Number of Patients by Gender/10-year Age Group. Accessed November 21, 2024 (https://opendata.hira.or.kr) |
|  |  | Ministry of Employment and Labor (MOEL, 2021). Survey Report on Labor Conditions by Employment Type. Accessed November 19, 2024 (https://kosis.kr) |

HIRA, Health Insurance Review and Assessment Service; MOEL, Ministry of Employment and Labor; NECA, National Evidence-based Healthcare Collaborating Agency; NPS, National Patient Sample

**Supplementary Table 2. Details on main analysis**

|  |  | **PPT** | **PT** |
| --- | --- | --- | --- |
| **Cost** | Treatment | 878.99 | 430.77 |
|  | Consultation | 349.31 | 953.75 |
|  | Syndrome differentiation technique | 76.04 | - |
|  | Healthcare system perspective | 1,304.33 | 1,384.52 |
|  | Transportation | 18.87 | 48.41 |
|  | Time (narrow) | 802.57 | 1,857.25 |
|  | Time (broad) | 1,181.40 | 2,733.90 |
|  | Restricted societal perspective (narrow) | 2,125.77 | 3,290.18 |
|  | Restricted societal perspective (broad) | 2,504.60 | 4,166.83 |
|  | Productivity loss (narrow) | 23,634.34 | 28,671.77 |
|  | Productivity loss (broad) | 34,266.75 | 41,570.37 |
|  | Societal pespective (narrow) | 25,760.12 | 31,961.95 |
|  | Societal pespective (broad) | 36,771.35 | 45,737.20 |
| **QALY** | EQ-5D | 2.30 | 2.23 |
| **ICER** | ICER: Healthcare system perspective | Dominant  -1,145.57 | |
|  | ICER: Societal pespective (narrow) | Dominant -88,597.57 | |
| **NMB** | NMB: Healthcare system persepctive | 1,945.66 | |
|  | NMB: Societal perspective | 8,067.30 | |

ICER, incremental cost-effectiveness ratios; NMB, net monetary benefit; PPT, pharmacopuncture therapy; PT, physiotherapy; QALY, quality-adjusted life year

**Supplementary Table 3. Results for one-way sensitivity analysis.**

|  | **Sensitivity range** | **Comparison** | **Total  cost ($)** | **Incremental  cost ($)** | **Total effect  (QALY)** | **Incremental effect (QALY)** | **ICER  ($/QALY)** |
| --- | --- | --- | --- | --- | --- | --- | --- |
| Base-case (Healthcare system perspective) |  | PPT | 1304.33 | -80.19 | 2.295 | 0.07 | Dominant |
|  |  | PT | 1384.52 |  | 2.225 |  | -1145.57 |
| Discount rate | 3.5% | PPT | 1319.84 | -82.36 | 2.325 | 0.071 | Dominant |
|  |  | PT | 1402.2 |  | 2.255 |  | -1160.00 |
|  | 6% | PPT | 1281.86 | -77.05 | 2.252 | 0.069 | Dominant |
|  |  | PT | 1358.91 |  | 2.183 |  | -1116.6667 |
| Initial severity  distribution | Moderate pain rate LCL | PPT | 1301.27 | -78.77 | 2.295 | 0.07 | Dominant |
|  |  | PT | 1380.04 |  | 2.226 |  | -1125.2857 |
|  | Moderate pain rate UCL | PPT | 1307.39 | -81.62 | 2.296 | 0.07 | Dominant |
|  |  | PT | 1389.01 |  | 2.225 |  | -1166.00 |
| Time horizon | 1 year | PPT | 529.95 | 29.22 | 0.793 | 0.023 | 1293.609 |
|  |  | PT | 500.73 |  | 0.771 |  |  |
|  | 5 year | PPT | 2013.4 | -178.53 | 3.671 | 0.113 | Dominant |
|  |  | PT | 2191.93 |  | 3.558 |  | -1579.91 |
| PPT treatment cost | Minimum | PPT | 1204.13 | -180.39 | 2.295 | 0.07 | Dominant |
|  | 15.48 | PT | 1384.52 |  | 2.225 |  | -2577.00 |
|  | Maximum | PPT | 1963.58 | 579.06 | 2.295 | 0.07 | 8271.347 |
|  | 30.58 | PT | 1384.52 |  | 2.225 |  |  |
| PT treatement type ratio | Superficial Heat Therapy+Transcutaneous Electrical Nerve Stimulation or  Superficial Heat Therapy+Interferential Current Therapy 100% | PPT | 1304.33 | -38.18 | 2.295 | 0.07 | Dominant |
|  |  | PT | 1342.51 |  | 2.225 |  | -545.43 |
|  | Deep Heat Therapy(Ultrasound)+ Laser Therapy 100% | PPT | 1304.33 | -224.15 | 2.295 | 0.07 | Dominant |
|  |  | PT | 1528.48 |  | 2.225 |  | -3202.14 |
|  | Deep Heat Therapy(Ultrasound)+Transcutaneous Electrical Nerve Stimulation  or Deep Heat Therapy(Ultrasound)+Interferential Current Therapy 100% | PPT | 1304.33 | -68.32 | 2.295 | 0.07 | Dominant |
|  |  | PT | 1372.65 |  | 2.225 |  | -976.00 |
|  | Superficial Heat Therapy+Laser Therapy 100% | PPT | 1304.33 | -194.01 | 2.295 | 0.07 | Dominant |
|  |  | PT | 1498.34 |  | 2.225 |  | -2771.57 |
| QALY  95% CI | Mild pain LCL | PPT | 1304.33 | -80.19 | 2.249 | 0.04 | Dominant |
|  |  | PT | 1384.52 |  | 2.209 |  | -2004.75 |
|  | Mild pain UCL | PPT | 1304.33 | -80.19 | 2.342 | 0.1 | Dominant |
|  |  | PT | 1384.52 |  | 2.242 |  | -801.90 |
|  | Moderate pain LCL | PPT | 1304.33 | -80.19 | 2.277 | 0.09 | Dominant |
|  |  | PT | 1384.52 |  | 2.188 |  | -891.00 |
|  | Moderate pain UCL | PPT | 1304.33 | -80.19 | 2.314 | 0.05 | Dominant |
|  |  | PT | 1384.52 |  | 2.263 |  | -1603.80 |
|  | Severe pain LCL | PPT | 1304.33 | -80.19 | 2.288 | 0.076 | Dominant |
|  |  | PT | 1384.52 |  | 2.212 |  | -1055.13 |
|  | Severe pain UCL | PPT | 1304.33 | -80.19 | 2.303 | 0.064 | Dominant |
|  |  | PT | 1384.52 |  | 2.239 |  | -1252.97 |
| Restricted societal perspective | Narrow | PPT | 2125.77 | -1164.4 | 2.295 | 0.07 | Dominant |
|  |  | PT | 3290.18 |  | 2.225 |  | -16634.29 |
|  | Broad | PPT | 2504.6 | -1662.23 | 2.295 | 0.07 | Dominant |
|  |  | PT | 4166.83 |  | 2.225 |  | -23746.14 |
| Societal System Perspective | Narrow | PPT | 25760.12 | -6201.83 | 2.295 | 0.07 | Dominant |
|  |  | PT | 31961.95 |  | 2.225 |  | -88597.57 |
|  | Broad | PPT | 36771.35 | -8965.85 | 2.295 | 0.07 | Dominant |
|  |  | PT | 45737.2 |  | 2.225 |  | -128083.57 |
| Transition probability  (ALL group,  95% CI) | Mild to Mild LCL | PPT | 1626.49 | 144.52 | 2.279 | 0.063 | 2286.34 |
|  |  | PT | 1481.97 |  | 2.216 |  |  |
|  | Mild to Mild UCL | PPT | 840.36 | -362.16 | 2.319 | 0.076 | Dominant |
|  |  | PT | 1202.52 |  | 2.243 |  | -4765.26 |
|  | Mild to Moderate LCL | PPT | 841.72 | -361.36 | 2.319 | 0.076 | Dominant |
|  |  | PT | 1203.08 |  | 2.243 |  | -4754.74 |
|  | Mild to Moderate UCL | PPT | 1625.84 | 144.03 | 2.279 | 0.063 | 2276.8 |
|  |  | PT | 1481.81 |  | 2.216 |  |  |
|  | Mild to Severe LCL | PPT | 1302.11 | -81.7 | 2.296 | 0.07 | Dominant |
|  |  | PT | 1383.81 |  | 2.226 |  | -1167.14 |
|  | Mild to Severe UCL | PPT | 1332.05 | -61.3 | 2.293 | 0.069 | Dominant |
|  |  | PT | 1393.35 |  | 2.224 |  | -888.41 |
| Transition probability (PPT group,  95% CI | Moderate to Mild  LCL | PPT | 1854.61 | 470.09 | 2.264 | 0.038 | 12287.08 |
|  |  | PT | 1384.52 |  | 2.225 |  |  |
|  | Moderate to Mild UCL | PPT | 967.32 | -417.2 | 2.315 | 0.089 | Dominant |
|  |  | PT | 1384.52 |  | 2.225 |  | -4687.64 |
|  | Moderate to Moderate LCL | PPT | 1090.32 | -294.2 | 2.305 | 0.079 | Dominant |
|  |  | PT | 1384.52 |  | 2.225 |  | -3724.05 |
|  | Moderate to Moderate UCL | PPT | 1810.15 | 425.63 | 2.274 | 0.048 | 8803.762 |
|  |  | PT | 1384.52 |  | 2.225 |  |  |
|  | Moderate to Severe  LCL | PPT | 1191.78 | -192.74 | 2.306 | 0.08 | Dominant |
|  |  | PT | 1384.52 |  | 2.225 |  | -2409.25 |
|  | Moderate to Severe UCL | PPT | 1454.44 | 69.92 | 2.282 | 0.056 | 1239.94 |
|  |  | PT | 1384.52 |  | 2.225 |  |  |
|  | Severe to Mild LCL | PPT | 1397.94 | 13.42 | 2.29 | 0.065 | 206.58 |
|  |  | PT | 1384.52 |  | 2.225 |  |  |
|  | Severe to Mild UCL | PPT | 1220.22 | -164.31 | 2.3 | 0.075 | Dominant |
|  |  | PT | 1384.52 |  | 2.225 |  | -2190.80 |
|  | Severe to Moderate  LCL | PPT | 1224.77 | -159.76 | 2.299 | 0.074 | Dominant |
|  |  | PT | 1384.52 |  | 2.225 |  | -2158.92 |
|  | Severe to Moderate  UCL | PPT | 1386.35 | 1.83 | 2.291 | 0.066 | 27.64 |
|  |  | PT | 1384.52 |  | 2.225 |  |  |
|  | Severe to Severe LCL | PPT | 1284.72 | -99.8 | 2.297 | 0.072 | Dominant |
|  |  | PT | 1384.52 |  | 2.225 |  | -1386.11 |
|  | Severe to Severe UCL | PPT | 1328.36 | -56.16 | 2.293 | 0.068 | Dominant |
|  |  | PT | 1384.52 |  | 2.225 |  | -825.88 |
| Transition probability (PT group,  95% CI | Moderate to Mild  LCL | PPT | 1304.33 | -205.39 | 2.295 | 0.083 | Dominant |
|  |  | PT | 1509.72 |  | 2.213 |  | -2474.58 |
|  | Moderate to Mild UCL | PPT | 1304.33 | 309.46 | 2.295 | 0.03 | 10159.31 |
|  |  | PT | 994.87 |  | 2.265 |  |  |
|  | Moderate to Moderate LCL | PPT | 1304.33 | 110.09 | 2.295 | 0.062 | 1789.83 |
|  |  | PT | 1194.24 |  | 2.234 |  |  |
|  | Moderate to Moderate UCL | PPT | 1304.33 | -331.72 | 2.295 | 0.081 | Dominant |
|  |  | PT | 1636.05 |  | 2.215 |  | -4095.31 |
|  | Moderate to Severe  LCL | PPT | 1304.33 | -121.05 | 2.295 | 0.062 | Dominant |
|  |  | PT | 1425.38 |  | 2.233 |  | -1952.42 |
|  | Moderate to Severe UCL | PPT | 1304.33 | -37.57 | 2.295 | 0.078 | Dominant |
|  |  | PT | 1341.9 |  | 2.218 |  | -481.67 |
|  | Severe to Mild LCL | PPT | 1304.33 | -219.54 | 2.295 | 0.084 | Dominant |
|  |  | PT | 1523.87 |  | 2.211 |  | -2613.57 |
|  | Severe to Mild UCL | PPT | 1304.33 | 36.18 | 2.295 | 0.058 | 624.38 |
|  |  | PT | 1268.15 |  | 2.238 |  |  |
|  | Severe to Moderate  LCL | PPT | 1304.33 | 9.74 | 2.295 | 0.063 | 154.56 |
|  |  | PT | 1294.59 |  | 2.232 |  |  |
|  | Severe to Moderate  UCL | PPT | 1304.33 | -162.42 | 2.295 | 0.076 | Dominant |
|  |  | PT | 1466.75 |  | 2.219 |  | -2137.11 |
|  | Severe to Severe LCL | PPT | 1304.33 | -69.72 | 2.295 | 0.067 | Dominant |
|  |  | PT | 1374.05 |  | 2.228 |  | -1040.60 |
|  | Severe to Severe UCL | PPT | 1304.33 | -93.15 | 2.295 | 0.074 | Dominant |
|  |  | PT | 1397.48 |  | 2.222 |  | -1258.78 |
